# Mutations in mitochondrial *ATAD3* gene and disease, lessons from *in vivo* models

**DOI:** 10.3389/fnins.2024.1496142

**Published:** 2024-11-13

**Authors:** Marcel Brügel, Ann-Sophie Kiesel, Tobias B. Haack, Susana Peralta

**Affiliations:** Institute of Medical Genetics and Applied Genomics, University of Tübingen, Tübingen, Germany

**Keywords:** *ATAD3*, cholesterol, mtDNA depletion and deletion, neurodegeneration, animal model, mitochondrial disease

## Abstract

Pathogenic variants in the *ATAD3* gene cluster have been associated with different neurodevelopmental disorders showing clinical symptoms like global developmental delay, muscular hypotonia, cardiomyopathy, congenital cataracts, and cerebellar atrophy. *ATAD3A* encodes for a mitochondrial ATPase whose function is unclear and has been considered one of the five most common nuclear genes associated with mitochondrial diseases in childhood. However, the mechanism causing *ATAD3-*associated disorders is still unknown. *In vivo* models have been used to identify *ATAD3* function. Here we summarize the features of mouse models with *ATAD3* loss of function and *Drosophila* models overexpressing pathogenic *ATAD3* variants. We discuss how these models have contributed to our understanding of *ATAD3* function and the pathomechanism of the *ATAD3-*associated disease.

## Introduction

ATPase family AAA domain-containing protein 3 (ATAD3) is a mitochondrial membrane protein from the family of ATPases associated with diverse cellular activities and conserved in metazoans. *ATAD3* gene absence results in embryonic lethality in worms (Hoffmann et al., 2009), flies (Gilquin et al., 2010; Harel et al., 2016), and mice (Goller et al., 2013; Peralta et al., 2018), indicating that it plays a role in early developmental stages and may be essential for proper mitochondrial function. In hominids, *ATAD3* has been duplicated twice to form an array of three paralog genes organized in tandem close to the telomere in chromosome 1p: *ATAD3A*, *ATAD3B*, and *ATAD3C*, whereas other species, such as fruit flies and mice, harbor only one gene (Li et al., 2014). *ATAD3B* differs from the ancestral paralog *ATAD3A* by having a C-terminal extension of 62 amino acids, which is caused by a mutation in the original stop codon, while *ATAD3C* seems to be a truncated gene, missing the first 70 amino acids of the protein (Li and Rousseau, 2012; Merle et al., 2012).

**Figure 1:**
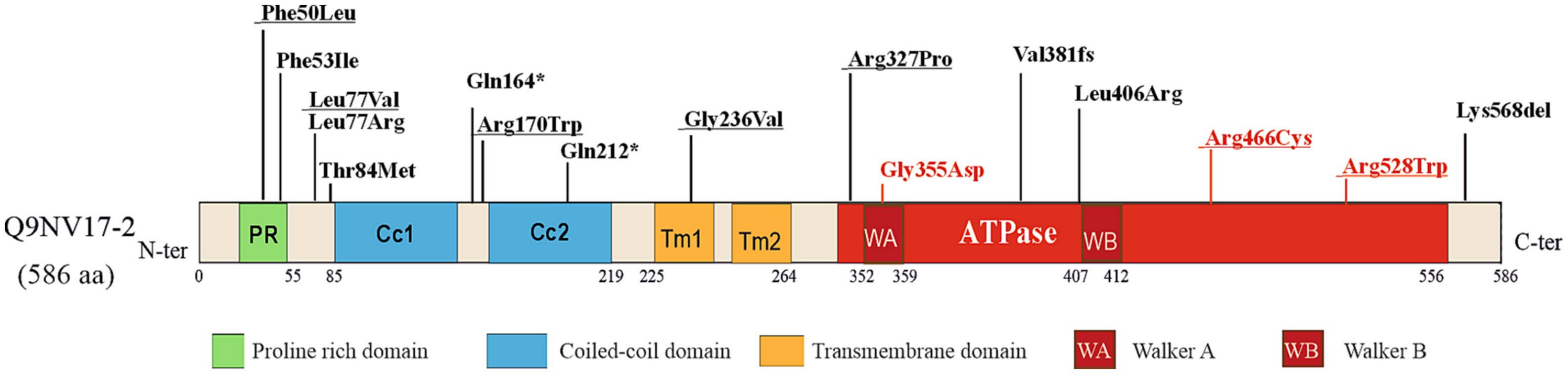
Single Nucleotide Pathogenic variants of human *ATAD3A*. Schematic representation of the human isoform 2 ATAD3A protein labeled with the main functional domains (NM_001170535.2, Q9NV17–2). PR stands for the proline-rich-domain and CC for the coiled-coil domain. The transmembrane (TM) domains are shown in orange, the ATPase domain in red, and the Walker A motif and Walker B motifs are denoted with WA and WB, respectively. The recessive and biallelic variants are marked by black arrows, while dominant variants are marked by red arrows. The variants that have been overexpressed in *Drosophila* and have been described in Table 1 are underlined.

ATAD3A has been described within mitochondria as spanning both mitochondrial membranes with its C terminus facing the matrix and the N-terminal region in the outer membrane (Gilquin et al., 2010; Baudier, 2018). The N-terminal domain comprises two transmembrane domains (TM1 and TM2), two coiled-coil domains (Cc1 and Cc2) important for protein–protein interactions and ATAD3A oligomerization, and a proline-rich domain (PR) of unknown function (Hubstenberger et al., 2010). The C-terminal region of ATAD3A contains an ATPase domain in the mitochondrial matrix with two conserved Walker A and Walker B motifs for ATP binding and ATPase activity (Figure 1). As a member of the AAA+ ATPase family, ATAD3 is predicted to form hexameric ring structures (Frickey and Lupas, 2004).

**Table 1:** *Drosophila* and mouse models developed to study the role of ATAD3.

	Genetic manipulation	Domain	Nature	Phenotype/Results	Citation
*Drosophila melanogaster*	Whole body knockout		Loss of function	Embryonic /L1 lethality Decreased mitochondrial content	Harel et al. (2016)
	Overexpression of WT			Larger and elongated mitochondria	
	Overexpression of the human variant: c.1582C > T (p.Arg528Trp) Drosophila R534W	ATPase	Dominant negative/gain of function	Decreased mitochondrial content. Disrupted mitochondrial morphology Increased autophagy	
	Whole body knockout (CRISPR/Cas9)		Loss of function	Severe neurodevelopmental defects in Drosophila embryos	Yap et al. (2021)
	Overexpression of the human variants: c.150C > G (p.Phe50Leu) Drosophila F56L	PR	Loss of function	Abnormal increase in mitochondrial content and size in embryos	
	c.229C > G (p.Leu77Val) Drosophila L83V	N-ter	Partial loss of function/Hypomorph	In adult thorax muscles: Small mitochondria with bar-shaped cristae and cristae abnormalities Increased autophagic intermediates	
	c.508C > T (p.Arg170Trp) Drosophila R176W	CC	Partial loss of function/Hypomorph		
	c.707G > T (p.Gly236Val) Drosophila G242V	TM	Loss of function	Abnormal increase in mitochondrial content and size in embryos	
	c.980G > C (p.Arg327Pro) Drosophila R333P	ATPase	Loss of function	Abnormal increase in mitochondrial content and size in embryos	
	c.1396C > T (p.Arg466Cys) Drosophila R472C	ATPase	Dominant negative/ gain of function	In Neuroblasts: Increased cholesterol in cellular membranes Increased lysosomal content	Muñoz-Oreja et al. (2024)
*Mus musculus*	Whole body knockout		Loss of function	Embryonic lethality	Goller et al. (2013), Peralta et al. (2018)
	Conditional knockout in skeletal muscle (Mlc1f-Cre)		Loss of function	Progressive Myopathy: Disruption of mitochondrial CJs and cristae morphology Reduced cristae surface and reduced mitochondrial size Cores negative for COX and SDH in muscle fibers Altered cholesterol metabolism mtDNA replication stalling mtDNA depletion and deletions Increased FGF21 in serum	Peralta et al. (2018)
	Conditional knockout in forebrain neurons (CaMKIIα-Cre)		Loss of function	Fatal progressive Neuropathy: Disruption of mitochondrial cristae morphology Reduced cristae surface and reduced mitochondrial size Disruption of lipid metabolism Accumulation of lipid droplets in the forebrain mtDNA depletion	Arguello et al. (2021)

Functionally, ATAD3A has been associated with several roles within mitochondria including regulation of the inner membrane structure, protein assembly (Peralta et al., 2018; Arguello et al., 2021), mitochondrial DNA (mtDNA) nucleoid organization (He et al., 2007; Gerhold et al., 2015; Desai et al., 2017), cholesterol trafficking, and lipid metabolism (Issop et al., 2015) among others (van den Ecker et al., 2015; Fang et al., 2010; Lang et al., 2020; Jin et al., 2018). However, the primary role of the protein remains unknown.

## *ATAD3A* variants and their associated diseases

*ATAD3A* is considered one of the five most common nuclear genes associated with mitochondrial diseases in childhood (Frazier et al., 2021). Due to the extensive sequence homology among the paralogs *ATAD3A*, *ATAD3B*, and *ATAD3C*, the 1p36.33 region is prone to non-allelic homologous recombination (NAHR), resulting in copy number variants (CNVs). Disease-causing mutations in *ATAD3A* include duplications and deletions among the *ATAD3* paralogs (Harel et al., 2016; Desai et al., 2017; Peeters-Scholte et al., 2017; Gunning et al., 2020; Frazier et al., 2021; Ebihara et al., 2022; Tawfik et al., 2023) and single nucleotide variants (SNVs) in the highly expressed *ATAD3A* gene (Cooper et al., 2017; Peralta et al., 2019; Dorison et al., 2020; Hanes et al., 2020; Muñoz-Oreja et al., 2024; Al Madhoun et al., 2019; Chen et al., 2023). The allelic spectrum of *ATAD3A*-associated diseases includes null, hypomorphic, and antimorphic alleles and the variants can be inherited and *de novo*. The SNVs reported to date in *ATAD3A* are depicted in Figure 1.

In 2016, pathogenic variants in *ATAD3A* were first associated with Harel-Yoon syndrome (MIM:617183), characterized by global developmental delay and muscular hypotonia, along with other features such as hypertrophic cardiomyopathy, optic atrophy, congenital cataracts, and peripheral neuropathy (Harel et al., 2016). Since then, *ATAD3A* variants have been associated with different neurodevelopmental disorders. The dominantly inherited heterozygous variant c.1064G > A (p.Gly355Asp) in the Walker A domain of ATAD3A was associated with hereditary spastic paraplegia and axonal neuropathy (Cooper et al., 2017). The heterozygous c.1396.C > T (p.Arg466Cys) missense variant, involved in the ATP hydrolysis, produces a form of neurological syndrome associated with optic atrophy (Muñoz-Oreja et al., 2024). Bi-allelic deletions of *ATAD3* via NAHR and compound heterozygous variants (some combinations of deletions/truncating mutations with missense mutations) have been associated with neonatal lethal pontocerebellar hypoplasia, hypotonia, and respiratory insufficiency syndrome (PHRINL, MIM618810; Desai et al., 2017; Peeters-Scholte et al., 2017; Skopkova et al., 2023). Moreover, a dominant 68 kilobase (kb) *de novo* duplication in the *ATAD3* locus was reported in 22 patients from 21 families, associated with a severe multisystemic disorder characterized by neonatal respiratory insufficiency, hypotonia, and cardiomyopathy, resulting in death in the first weeks of life (MIM:618815; Gunning et al., 2020; Frazier et al., 2021). These duplications generate an extra copy of *ATAD3B* and an in-frame *ATAD3A/C* fusion gene that forms a stable chimeric ATAD3A/C protein disrupting regular ATAD3 oligomerization. The extra copy of *ATAD3B* is not thought to play a role in the phenotype as healthy individuals and patients with distinct phenotypes were found to have benign duplications affecting only *ATAD3B*.

## *Drosophila* models overexpressing pathogenic *ATAD3* variants

To functionally evaluate the potential pathogenicity of the *ATAD3A* variants several transgenic flies have been generated using the *UAS-*Gal4 system for tissue-specific expression (Table 1). The *Drosophila melanogaster* ortholog to the human *ATAD3A* is called *Belphegor* (*bor*), hereafter referred to as “*Drosophila ATAD3*” or “*dATAD3*.”

In 2016, Harel et al. studied the recurrent heterozygous *de novo* variant c.1582C > T (p.Arg528Trp), located in the ATPase domain of the protein (equivalent in *Drosophila*, *p.R534W*; Figure 1; Harel et al., 2016). This variant was found in five unrelated families associated with global developmental delay, axonal neuropathy, and hypertrophic cardiomyopathy. They demonstrated that ubiquitous (*tub*-Gal4 and *Ubi*-Gal4), pan-neuronal (*n-syb*-Gal4), and motoneuronal (*D42*-Gal4) expression of UAS-*dAtad3^R534W^* resulted in complete embryonic lethality with no viable adult flies. Muscle-specific expression (*C57*-Gal4) led to approximately 90% lethality. In contrast, overexpression of the wild-type allele UAS-*dAtad3^WT^* with the same Gal4 drivers consistently produced viable flies with no phenotype. On a cellular level, the *p.R534W* mutation induced a significant reduction of mitochondria in the ventral nerve cord, axons, and synaptic boutons, suggesting increased mitophagy. Transmission electron microscopy (TEM) showed that muscle tissue contained very few and small mitochondria with highly aberrant cristae and a substantial increase in autophagic intermediates. Overexpression of *dAtad3^WT^*, however, resulted in the opposite phenotype, with large, elongated mitochondria. The authors proposed that *ATAD3* may promote mitochondrial fusion or inhibit fission, while the mutation likely inhibits fusion and/or promotes fission.

In 2021, Yap et al. investigated five *ATAD3A* missense variants inherited in trans to loss-of-function (LOF) alleles and associated with distinct neurological phenotypes (Yap et al., 2021). The variants included c.150C > G (p.Phe50Leu), c.229C > G (p.Leu77Val), in the N-terminal of ATAD3A, c.508C > T (p.Arg170Trp) located between the coiled-coil domains; c.707G > T (p.Gly236Val) in the transmembrane domain, and c.980G > C (p.Arg327Pro) in the ATPase domain (Figure 1). The equivalent variants in *Drosophila* are *dAtad3^F56L^*, *dAtad3^L83K^*, *dAtad3^R176W^*, *dAtad3^G242V^* and *dAtad3^R333P^* (Table 1). Using CRISPR/Cas9-mediated genome editing, the researchers integrated a gene cassette into the first intron of *dAtad3* generating a LOF allele. All flies harboring this LOF allele and a null allele (PBac{*PB*}*dAtad3a^c05496^*), resulted in a functional knockout. The lethality was rescued by UAS-*dAtad3^WT^* expression using a pan-neuronal Gal4 driver (elav^C155^-Gal4), confirming the severe LOF nature of the introduced cassette. The expression of *dAtad3^L83V^* and *dAtad3^R176W^* variants also rescued the developmental lethality caused by *ATAD3A* loss, suggesting that they are partial LOF alleles. The other 3 variants, *dAtad3^F56L^*, *dAtad3^G242V^*, and *dAtad3^R333P^* failed to rescue lethality indicating that they are severe LOF alleles.

Further characterization of the *dAtad3^L83V^* and *dAtad3^R176W^* variants showed a decreased lifespan, and locomotion and flight defects. TEM analysis showed both mutations caused small mitochondria with cristae abnormalities and increased autophagic intermediates. Confocal images showed increased expression of the autophagic marker p62 in adult thorax muscles expressing *dAtad3^L83V^* and *dAtad3^R176W^*. The authors concluded that ATAD3A function is required for the homeostasis of mitochondrial dynamics and mitophagy. One possible mechanism proposed was through increased interaction with Drp1, as the coiled-coil domain was shown to interact with Drp1 promoting mitochondrial fission (Zhao et al., 2019). Moreover, this study revealed the functional importance of the N-terminal, coiled-coil, and transmembrane domains of ATAD3A.

Recently, Muñoz-Oreja et al. investigated the heterozygous p.Arg466Cys variant (*p.R472C* in *Drosophila*), affecting a conserved arginine finger crucial for ATP hydrolysis (Figure 1). Transgenic flies with UAS-*dAtad3*^R472C^ were created and crossed with various tissue-specific Gal4 drivers (Muñoz-Oreja et al., 2024). Ubiquitous expression resulted in complete lethality, likely through a dominant-negative mechanism. Expression in nervous and muscle tissue (*Atad3-*Gal4) or neurons (*elav^C155^-*Gal4) was lethal as well. However, expression driven by *eyeless*-Gal4 (*ey-*Gal4), which is limited to the eyes and part of the brain, resulted only in partial lethality (65%), with the surviving flies exhibiting abnormal or missing eyes. Moreover, using the neuroblast-specific *inscuteable*-Gal4 (*insc*-Gal4) driver or the late-onset eye and neuronal driver *glass multiple reporter*-Gal4 (*GMR*-Gal4), viable flies expressing the mutant variant dAtad3^R472C^ were produced similarly to controls. Therefore, the Arg466Cys variant is highly deleterious unless expression is highly restricted. Expression of the p.Arg466Cys variant in neuroblasts led to the formation of membrane-bound cholesterol aggregates and increased lysosomal content. The cholesterol aggregates, detected by the reporter mKate-D4, co-localized with the lysosomal marker LAMP-GFP, suggesting that this excess of cholesterol is targeted to the lysosomal pathway. In agreement with the results obtained in *Drosophila*, patient-derived fibroblasts also exhibited membrane-embedded cholesterol aggregation in the form of membrane whorls and increased lysosomal content. Interestingly, flies expressing the p.Arg466Cys variant under the *ey*-Gal4 driver showed higher dependency on dietary cholesterol. A diet with reduced cholesterol significantly decreased the number of viable adults, and, by contrast, cholesterol supplementation on the diet enhanced viability. Based on these results, the authors propose a model where defective ATAD3 results in a mitochondrial cholesterol deficit that is attenuated by increasing the cytosolic cholesterol levels. This increased cholesterol would be a cellular compensatory response that leads to an aberrant aggregation in membranes that can cascade to lysosomal insufficiency contributing to the pathomechanism of the disease.

## Mouse models of *ATAD3* loss of function

In mice, the ubiquitous disruption of ATAD3 was embryonic lethal (Peralta et al., 2018; Goller et al., 2013). To understand the *in vivo* function of ATAD3 in mammals we generated two different LOF mouse models: the ATAD3 skeletal muscle deficient mice (Peralta et al., 2018) and ATAD3-neuron deficient mice (Arguello et al., 2021). The main features observed in the animal models are summarized in Table 1.

### *ATAD3* skeletal muscle knockout

*ATAD3* skeletal muscle-deficient mice (*ATAD3* muscle KO) were obtained by crossing the *ATAD3* floxed with Mlc1f-Cre transgenic mice, expressing Cre recombinase under the myosin light chain 1 (Mlc1) promoter (Bothe et al., 2000). The lack of *ATAD3* in skeletal muscle induced a progressive myopathy with an onset between 2 and 3 months of age, characterized by motor-impaired coordination and weakness, developing into muscle wasting, and reduced fiber size (Peralta et al., 2018). Despite the dramatically reduced muscle tonus, *ATAD3* muscle KO mice did not show reduced survival. This fact suggests that skeletal muscle mitochondria are either able to compensate for the resulting functional consequences, or that in skeletal muscle, *ATAD3* is not essential for general survival.

The first phenotype detected by TEM in the muscles of 2-month-old *ATAD3* KO mice was a disruption of the inner mitochondrial membrane (IMM) structure. The cristae, formed by the inner membrane, lost the cristae junctions (CJs), and the cristae surface per mitochondria was decreased (Table 1). This was accompanied by a reduction in high molecular weight mitochondrial contact site and cristae organizing system (MICOS) complexes. As degeneration of the cristae increased over time, in the muscles from 5-month-old animals, the CJs and the lamellar structure of the cristae were mostly absent with predominant circular structures. In addition, *ATAD3* KO muscles had reduced mitochondrial size. These findings indicated that *ATAD3* is required for the integrity of mitochondrial cristae in skeletal muscle.

Furthermore, the lack of muscular *ATAD3* resulted in mtDNA replication stalling (indicated by the accumulation of replication intermediates), causing progressive mtDNA depletion and deletions in the KO muscle. Indeed, the levels of the myokine FGF21, a biomarker for mtDNA-related myopathies (Lehtonen et al., 2016), were increased in *ATAD3* KO muscle compared to controls. This result suggested that mtDNA replication might be coupled with cristae organization.

Lipidomic studies showed that *ATAD3* KO muscles had decreased levels of cholesterol esters (CEs) synthesized in the ER (generally containing short saturated or monounsaturated acyl chains) and increased levels of CEs obtained from the diet (generally containing longer and more unsaturated acyl chains). A 30% decrease in the ratio of total CEs versus free cholesterol (unesterified cholesterol) was detected in the KO muscles of 5-month-old mice when most of the mitochondria had disrupted cristae. These results indicated that cholesterol is internalized but does not reach the mitochondria, hinting at a cholesterol-trafficking defect. As cholesterol-rich membrane structures are important for tethering mtDNA nucleoids to the inner mitochondrial membranes (Gerhold et al., 2015), these results link *ATAD3* to cholesterol-dependent cristae organization and mtDNA maintenance.

Interestingly, in the immunohistochemistry staining the muscle fibers of the KO tissues presented pale cores that were negative for both COX (cytochrome oxidase) and SDH (succinate dehydrogenase) activities, indicating reduced mitochondrial mass in focal areas. This suggests that *ATAD3* has a key role in the preservation of the mitochondrial network in muscle. Oxidative phosphorylation (OXPHOS) activity within muscle fibers in the KO mice was not severely affected. Only the complexes known to be dependent on cristae structure, such as complex V, and supercomplexes containing complex I and III were reduced in the KO muscles. These results demonstrate that *ATAD3* does not have a significant role in mitochondrial translation, as previously suggested (He et al., 2012) and that it is not crucial for OXPHOS assembly. *ATAD3* KO skeletal muscles presented increased PGC-1α and SDHA levels, probably as a result of the induction of mitochondrial biogenesis as a compensatory mechanism.

Altogether, these results indicated a critical early role of *ATAD3* in regulating IMM structure, leading to secondary defects in cholesterol homeostasis, mtDNA replication, and OXPHOS levels in muscle tissue.

### *ATAD3* neuron knockout

*ATAD3*-neuron deficient mice (*ATAD3* neuron KO) were obtained by crossing the *ATAD3* floxed mice with CaMKIIα transgenic mice, expressing Cre recombinase under the calcium/calmodulin-dependent protein kinase II (CaMKIIα) promoter. The CaMKIIα gene is expressed predominantly in the cortex and hippocampus neurons (Dragatsis and Zeitlin, 2000). The lack of *ATAD3* in forebrain neurons resulted in a fatal progressive encephalopathy with an onset at 5 months old (Arguello et al., 2021). The *ATAD3* neuron KO mice showed symptoms such as impaired motor coordination and disrupted stereotypical rodent behavior that worsened with time. Contrary to what was observed in the muscle model, ablation of *ATAD3* in neurons resulted in premature death, indicating that *ATAD3* function in neurons is essential for survival.

Coinciding with the muscle model, the first phenotype detected in the *ATAD3* neuron KO model was a disrupted cristae morphology in the hippocampus region of pre-symptomatic KO mice of 2 months old. Quantification analysis of the TEM images demonstrated that *ATAD3* KO neurons had reduced mitochondrial size and reduced cristae surface per mitochondria. The mtDNA levels were similar in *ATAD3* neuron KO and control tissues of 3 months old (pre-symptomatic stage). However, 2 months later, mtDNA levels were decreased in *ATAD3* neuron KO cortical and hippocampal areas. Neuronal cell death and decreased OXPHOS levels were detected only in 5-month-old tissues, concurring with the mtDNA depletion.

Metabolomics and lipidomic analysis performed in *ATAD3* neuron KO mice at the pre-symptomatic stage revealed altered pathways related to the transport of lipids along mitochondria membranes, for example in the carnitine shuttle pathway, which transports long fatty acid chains through the IMM. Also, several precursors of the cardiolipin synthesis pathway, the main fatty acyl moiety in mitochondria, and phosphatidylcholine, one of the most abundant phospholipids in both mitochondrial membranes, were decreased.

Overall, these results indicated a role of *ATAD3* in the preservation of the cristae morphology and mitochondrial lipid metabolism in neurons. This results over the months in mtDNA depletion and neuron cell death.

## Discussion and concluding remarks

Animal models for *ATAD3*/*Atad3* dysfunction have become indispensable for studying *ATAD3* functions *in vivo* and effectively recapitulate many features observed in patients’ cells with *ATAD3* variants. In the last years, several advanced techniques have been used to study these features.

High-resolution approaches like TEM have provided critical insights into mitochondrial cristae structure. Both animal models and patient cells show disrupted mitochondrial cristae morphology (Peralta et al., 2019; Dorison et al., 2020) and mitochondrial fragmentation (Cooper et al., 2017). This aligns with ATAD3A’s interaction with other proteins like PROHIBITIN and LETM1, that are essential for maintaining cristae morphology (Arguello et al., 2021; Antonicka et al., 2020). Also, in human embryonic kidney (HEK) cells, ATAD3A showed a remarkably regular distribution across the mitochondrial membrane, a typical characteristic of scaffolding proteins (Arguello et al., 2021).

Furthermore, fluorescence microscopy plays a crucial role in the analysis of cholesterol metabolism. In patient cells harboring ATAD3 gene cluster deletions, or duplication resulting in the formation of an ATAD3A/C fusion protein, elevated levels of unesterified cholesterol have been identified by filipin staining (Desai et al., 2017; Gunning et al., 2020). The animal models for *ATAD3* dysfunction also reflect patient findings in terms of altered lipid metabolism. In 2024, a study using the *Drosophila* model introduced the novel application of the cholesterol reporter mKate-D4, which enabled the detection of membrane-bound cholesterol *in vivo* (Muñoz-Oreja et al., 2024). This novel approach facilitates to detect this difficult to study area of the cell metabolism, the cholesterol metabolism, which has been previously implicated in the disease. The researchers demonstrated that *Atad3* dysfunction leads to a compensatory increase in cellular cholesterol, resulting in its abnormal aggregation in membranes that can cascade into lysosomal insufficiency, which may contribute to the pathomechanism of the disease.

Aberrant organization of mtDNA was observed in *ATAD3* patients’ fibroblasts by immunocytochemistry staining (Desai et al., 2017; Gunning et al., 2020; Muñoz-Oreja et al., 2024), but has not been studied in animal models. However, in mouse models, mtDNA depletion occurs after cristae disorganization, suggesting that *ATAD3* affects mtDNA maintenance *in vivo* by stabilizing the mitochondrial cristae morphology (Peralta et al., 2018; Arguello et al., 2021).

As previously described, *ATAD3* LOF in skeletal muscle tissue of mice resulted in a milder phenotype than the neuron model. This result highlights the existence of different compensatory mechanisms to counteract *ATAD3* dysfunction in different tissues. Indeed, Frazier et al. also indicated this tissue specificity, where complex I activity was more profoundly reduced in cardiac tissue than in skeletal muscle or fibroblasts from *ATAD3*-deficient patients (Frazier et al., 2021).

However, species-specific variations observed in the *ATAD3A* gene structure present a potential limitation in the direct application of findings from animal models to human disease. To illustrate, consider the case of the NAHR-mediated duplication syndrome, which is characterized by pontocerebellar hypoplasia, seizures, and respiratory insufficiency (Gunning et al., 2020; Frazier et al., 2021). On a molecular level, the duplications typically result in a stable chimeric ATAD3A/C protein harboring 29 missense changes, including the previously referenced p.Arg466Cys variant, which is postulated to be a significant contributor to pathogenicity. However, the p.Arg466Cys variant itself is associated with a milder phenotype including syndromic dominant optic atrophy with neurological involvement (Muñoz-Oreja et al., 2024). This suggests that the variant is only partially responsible for the phenotype observed in the duplication syndrome. Nevertheless, ubiquitous expression of the Arg466Cys variant in *Drosophila* proved to be lethal. Thus, the animal model, which lacks the *ATAD3B* and *ATAD3C* genes, is unable to fully elucidate the underlying pathomechanisms in this case.

Overall, the results obtained from the *Drosophila* and mouse models, along with other *in vitro* studies, have yielded valuable insights into the function of *ATAD3A*. In summary, it has been demonstrated that *ATAD3A* LOF or CNVs in the *ATAD3* locus result in mitochondrial dysfunction, due to the protein’s role in the structural organization of mitochondrial membranes and its impact on essential processes such as mtDNA maintenance and cholesterol metabolism. Nevertheless, the precise function of ATAD3A and the cellular mechanisms underlying *ATAD3-*associated disorders remain unclear and require further research. The integration of genomic, transcriptomic, proteomic, lipidomic, and metabolomic data, coupled with the application of novel advanced technologies, is a promising avenue for advancing our understanding of pathomechanisms and may also facilitate the identification of potential therapeutic targets.

## References

[ref1] Al Madhoun A. Alnaser F. Melhem M. Nizam R. Al-Dabbous T. Al-Mulla F. (2019). Ketogenic diet attenuates cerebellar atrophy progression in a subject with a biallelic variant at the Atad3A locus. Appl. Clin. Genet. 12, 79–86. doi: 10.2147/TACG.S194204, PMID: 31239750 PMC6556476

[ref2] Antonicka H. Lin Z. Y. Janer A. Aaltonen M. J. Weraarpachai W. Gingras A. C.. (2020). A high-density human mitochondrial proximity interaction network. Cell Metab. 32, 479–497.e9. doi: 10.1016/j.cmet.2020.07.017 32877691

[ref3] Arguello T. Peralta S. Antonicka H. Gaidosh G. Diaz F. Tu Y.-T.. (2021). Atad3A has a scaffolding role regulating mitochondria inner membrane structure and protein assembly. Cell Rep. 37:110139. doi: 10.1016/j.celrep.2021.110139, PMID: 34936866 PMC8785211

[ref4] Baudier J. (2018). Atad3 proteins: brokers of a mitochondria-endoplasmic reticulum connection in mammalian cells. Biol. Rev. Camb. Philos. Soc. 93, 827–844. doi: 10.1111/brv.12373, PMID: 28941010

[ref5] Bothe G. W. Haspel J. A. Smith C. L. Wiener H. H. Burden S. J. (2000). Selective expression of Cre recombinase in skeletal muscle fibers. Genesis 26, 165–166. doi: 10.1002/(SICI)1526-968X(200002)26:2<165:AID-GENE22>3.0.CO;2-F, PMID: 10686620

[ref6] Chen L. Li Y. Zambidis A. Papadopoulos V. (2023). Atad3A: a key regulator of mitochondria-associated diseases. Int. J. Mol. Sci. 24:12511. doi: 10.3390/ijms241512511 37569886 PMC10419812

[ref7] Cooper H. M. Yang Y. Ylikallio E. Khairullin R. Woldegebriel R. Lin K. L.. (2017). Atpase-deficient mitochondrial inner membrane protein Atad3A disturbs mitochondrial dynamics in dominant hereditary spastic paraplegia. Hum. Mol. Genet. 26, 1432–1443. doi: 10.1093/hmg/ddx042, PMID: 28158749 PMC5393146

[ref8] Desai R. Frazier A. E. Durigon R. Patel H. Jones A. W. Dalla Rosa I.. (2017). Atad3 gene cluster deletions cause cerebellar dysfunction associated with altered mitochondrial Dna and cholesterol metabolism. Brain 140, 1595–1610. doi: 10.1093/brain/awx094, PMID: 28549128 PMC5445257

[ref9] Dorison N. Gaignard P. Bayot A. Gelot A. Becker P. H. Fourati S.. (2020). Mitochondrial dysfunction caused by novel Atad3A mutations. Mol. Genet. Metab. 131, 107–113. doi: 10.1016/j.ymgme.2020.09.002, PMID: 32933822

[ref10] Dragatsis I. Zeitlin S. (2000). Camkiialpha-Cre transgene expression and recombination patterns in the mouse brain. Genesis 26, 133–135. doi: 10.1002/(SICI)1526-968X(200002)26:2<133:AID-GENE10>3.0.CO;2-V 10686608

[ref11] Ebihara T. Nagatomo T. Sugiyama Y. Tsuruoka T. Osone Y. Shimura M.. (2022). Severe spinal cord hypoplasia due to a novel Atad3A compound heterozygous deletion. Mol Genet Metab Rep 33:100912. doi: 10.1016/j.ymgmr.2022.100912, PMID: 36061954 PMC9428837

[ref12] Fang H. Y. Chang C. L. Hsu S. H. Huang C. Y. Chiang S. F. Chiou S. H.. (2010). Atpase family Aaa domain-containing 3A is a novel anti-apoptotic factor in lung adenocarcinoma cells. J. Cell Sci. 123, 1171–1180. doi: 10.1242/jcs.062034, PMID: 20332122

[ref13] Frazier A. E. Compton A. G. Kishita Y. Hock D. H. Welch A. E. Amarasekera S. S. C.. (2021). Fatal perinatal mitochondrial cardiac failure caused by recurrent de novo duplications in the Atad3 locus. Med (N Y) 2, 49–73. doi: 10.1016/j.medj.2020.06.004 PMC7875323 33575671

[ref14] Frickey T. Lupas A. N. (2004). Phylogenetic analysis of Aaa proteins. J. Struct. Biol. 146, 2–10. doi: 10.1016/j.jsb.2003.11.020 15037233

[ref15] Gerhold J. M. Cansiz-Arda Ş. Lõhmus M. Engberg O. Reyes A. Van Rennes H.. (2015). Human mitochondrial Dna-protein complexes attach to a cholesterol-rich membrane structure. Sci. Rep. 5:15292. doi: 10.1038/srep15292, PMID: 26478270 PMC4609938

[ref16] Gilquin B. Taillebourg E. Cherradi N. Hubstenberger A. Gay O. Merle N.. (2010). The Aaa+ Atpase Atad3A controls mitochondrial dynamics at the interface of the inner and outer membranes. Mol. Cell. Biol. 30, 1984–1996. doi: 10.1128/MCB.00007-10, PMID: 20154147 PMC2849464

[ref17] Goller T. Seibold U. K. Kremmer E. Voos W. Kolanus W. (2013). Atad3 function is essential for early post-implantation development in the mouse. PLoS One 8:e54799. doi: 10.1371/journal.pone.0054799, PMID: 23372768 PMC3556029

[ref18] Gunning A. C. Strucinska K. Muñoz Oreja M. Parrish A. Caswell R. Stals K. L.. (2020). Recurrent De novo Nahr reciprocal duplications in the Atad3 gene cluster cause a Neurogenetic trait with perturbed cholesterol and mitochondrial metabolism. Am. J. Hum. Genet. 106, 272–279. doi: 10.1016/j.ajhg.2020.01.007, PMID: 32004445 PMC7010973

[ref19] Hanes I. Mcmillan H. J. Ito Y. Kernohan K. D. Lazier J. Lines M. A.. (2020). A splice variant in Atad3A expands the clinical and genetic spectrum of Harel-Yoon syndrome. Neurol Genet 6:e452. doi: 10.1212/NXG.0000000000000452, PMID: 32607449 PMC7286657

[ref20] Harel T. Yoon W. H. Garone C. Gu S. Coban-Akdemir Z. Eldomery M. K.. (2016). Recurrent De novo and Biallelic variation of Atad3A, encoding a mitochondrial membrane protein, results in distinct neurological syndromes. Am. J. Hum. Genet. 99, 831–845. doi: 10.1016/j.ajhg.2016.08.007, PMID: 27640307 PMC5065660

[ref21] He J. Cooper H. M. Reyes A. Di Re M. Sembongi H. Litwin T. R.. (2012). Mitochondrial nucleoid interacting proteins support mitochondrial protein synthesis. Nucleic Acids Res. 40, 6109–6121. doi: 10.1093/nar/gks266, PMID: 22453275 PMC3401451

[ref22] He J. Mao C. C. Reyes A. Sembongi H. Di Re M. Granycome C.. (2007). The Aaa+ protein Atad3 has displacement loop binding properties and is involved in mitochondrial nucleoid organization. J. Cell Biol. 176, 141–146. doi: 10.1083/jcb.200609158, PMID: 17210950 PMC2063933

[ref23] Hoffmann M. Bellance N. Rossignol R. Koopman W. J. Willems P. H. Mayatepek E.. (2009). *C. elegans* Atad-3 is essential for mitochondrial activity and development. PLoS One 4:e7644. doi: 10.1371/journal.pone.0007644, PMID: 19888333 PMC2765634

[ref24] Hubstenberger A. Merle N. Charton R. Brandolin G. Rousseau D. (2010). Topological analysis of Atad3A insertion in purified human mitochondria. J. Bioenerg. Biomembr. 42, 143–150. doi: 10.1007/s10863-010-9269-8, PMID: 20349121

[ref25] Issop L. Fan J. Lee S. Rone M. B. Basu K. Mui J.. (2015). Mitochondria-associated membrane formation in hormone-stimulated Leydig cell steroidogenesis: role of Atad3. Endocrinology 156, 334–345. doi: 10.1210/en.2014-1503, PMID: 25375035

[ref26] Jin G. Xu C. Zhang X. Long J. Rezaeian A. H. Liu C.. (2018). Atad3a suppresses Pink1-dependent mitophagy to maintain homeostasis of hematopoietic progenitor cells. Nat. Immunol. 19, 29–40. doi: 10.1038/s41590-017-0002-1, PMID: 29242539 PMC5905408

[ref27] Lang L. Loveless R. Teng Y. (2020). Emerging links between control of mitochondrial protein Atad3A and Cancer. Int. J. Mol. Sci. 21:7917. doi: 10.3390/ijms21217917, PMID: 33113782 PMC7663417

[ref28] Lehtonen J. M. Forsström S. Bottani E. Viscomi C. Baris O. R. Isoniemi H.. (2016). Fgf21 is a biomarker for mitochondrial translation and mtdna maintenance disorders. Neurology 87, 2290–2299. doi: 10.1212/WNL.0000000000003374, PMID: 27794108 PMC5270510

[ref29] Li S. Lamarche F. Charton R. Delphin C. Gires O. Hubstenberger A.. (2014). Expression analysis of Atad3 isoforms in rodent and human cell lines and tissues. Gene 535, 60–69. doi: 10.1016/j.gene.2013.10.062 24239551

[ref30] Li S. Rousseau D. (2012). Atad3, a vital membrane bound mitochondrial Atpase involved in tumor progression. J. Bioenerg. Biomembr. 44, 189–197. doi: 10.1007/s10863-012-9424-5, PMID: 22318359

[ref31] Merle N. Féraud O. Gilquin B. Hubstenberger A. Kieffer-Jacquinot S. Assard N.. (2012). Atad3B is a human embryonic stem cell specific mitochondrial protein, re-expressed in cancer cells, that functions as dominant negative for the ubiquitous Atad3A. Mitochondrion 12, 441–448. doi: 10.1016/j.mito.2012.05.005, PMID: 22664726

[ref32] Muñoz-Oreja M. Sandoval A. Bruland O. Perez-Rodriguez D. Fernandez-Pelayo U. De Arbina A. L.. (2024). Elevated cholesterol in Atad3 mutants is a compensatory mechanism that leads to membrane cholesterol aggregation. Brain. 147, 1899–1913. doi: 10.1093/brain/awae018 38242545 PMC11068212

[ref33] Peeters-Scholte C. M. P. C. D. Adama Van Scheltema P. N. Klumper F. J. C. M. Everwijn S. M. P. Koopmans M. Hoffer M. J. V.. (2017). Genotype-phenotype correlation in Atad3A deletions: not just of scientific relevance. Brain 140:e66. doi: 10.1093/brain/awx239 29053797

[ref34] Peralta S. Goffart S. Williams S. L. Diaz F. Garcia S. Nissanka N.. (2018). Atad3 controls mitochondrial cristae structure in mouse muscle, influencing mtdna replication and cholesterol levels. J. Cell Sci. 131:217075. doi: 10.1242/jcs.217075, PMID: 29898916 PMC6051345

[ref35] Peralta S. González-Quintana A. Ybarra M. Delmiro A. Pérez-Pérez R. Docampo J.. (2019). Novel Atad3A recessive mutation associated to fatal cerebellar hypoplasia with multiorgan involvement and mitochondrial structural abnormalities. Mol. Genet. Metab. 128, 452–462. doi: 10.1016/j.ymgme.2019.10.012 31727539

[ref36] Skopkova M. Stufkova H. Rambani V. Stranecky V. Brennerova K. Kolnikova M.. (2023). Atad3A-related pontocerebellar hypoplasia: new patients and insights into phenotypic variability. Orphanet J. Rare Dis. 18:92. doi: 10.1186/s13023-023-02689-3, PMID: 37095554 PMC10127305

[ref37] Tawfik C. A. Zaitoun R. Farag A. A. (2023). Harel Yoon syndrome: a novel mutation in Atad3A gene and expansion of the clinical spectrum. Ophthalmic Genet. 44, 226–233. doi: 10.1080/13816810.2023.2183223 36856321

[ref38] Van Den Ecker D. Hoffmann M. Müting G. Maglioni S. Herebian D. Mayatepek E.. (2015). *Caenorhabditis elegans* Atad-3 modulates mitochondrial iron and heme homeostasis. Biochem. Biophys. Res. Commun. 467, 389–394. doi: 10.1016/j.bbrc.2015.09.143, PMID: 26427876

[ref39] Yap Z. Y. Park Y. H. Wortmann S. B. Gunning A. C. Ezer S. Lee S.. (2021). Functional interpretation of Atad3A variants in neuro-mitochondrial phenotypes. Genome Med. 13:55. doi: 10.1186/s13073-021-00873-3, PMID: 33845882 PMC8042885

[ref40] Zhao Y. Sun X. Hu D. Prosdocimo D. A. Hoppel C. Jain M. K.. (2019). Atad3A oligomerization causes neurodegeneration by coupling mitochondrial fragmentation and bioenergetics defects. Nat. Commun. 10:1371. doi: 10.1038/s41467-019-09291-x, PMID: 30914652 PMC6435701

